# Retrospective analysis of surgical and oncological results of laparoscopic surgeries performed by residents of coloproctology

**DOI:** 10.1590/0100-6991e-20233404-en

**Published:** 2023-05-11

**Authors:** BÁRBARA BIANCA LINHARES MOTA, TARCÍSIO JUNIOR BITTENCOURT MACEDO, ROGÉRIO SERAFIM PARRA, JOSÉ JOAQUIM RIBEIRO DA ROCHA, OMAR FERES, MARLEY RIBEIRO FEITOSA

**Affiliations:** 1 - Hospital das Clínicas da Faculdade de Medicina de RIbeirão Preto - USP, Departamento de anatomia e cirurgia, divisão de coloproctologia - Ribeirao Preto - SP - Brasil

**Keywords:** Colorectal Surgery, Laparoscopy, Education Medical, Cirurgia Colorretal, Laparoscopia, Educação Médica

## Abstract

**Introduction:**

with the improvement and wide acceptance of laparoscopy in colorectal operations, there was a need for specific training of surgeons in training. There are few studies evaluating the postoperative results of laparoscopic colectomies performed by resident physicians and their impact on patient safety.

**Purpose:**

to analyze the surgical and oncological results of laparoscopic colectomies performed by coloproctology residents and compare them with data in the literature.

**Methods:**

this is a retrospective analysis of patients undergoing laparoscopic colorectal surgery performed by resident physicians at the Hospital das Clínicas de Ribeirão Preto, between 2014 and 2018. The clinical characteristics of the patients were studied, as well as the main surgical and oncological aspects in a period of one year.

**Results:**

we analyzed 191 operations, whose main surgical indication was adenocarcinoma, most of them stage III. The mean duration of surgeries was 210±58 minutes. There was a need for a stoma in 21.5% of the patients, mainly loop colostomy. The conversion rate was 23%, with 79.5% due to technical difficulties, and the main predictors of conversion were obesity and intraoperative accidents. The median length of stay was 6 days. Preoperative anemia was associated with a higher rate of complications (11.5%) and reoperations (12%). Surgical resection margins were compromised in 8.6% of cases. The one-year recurrence rate was 3.2% and the mortality rate was 6.3%.

**Conclusions:**

videolaparoscopic colorectal surgery performed by residents showed efficacy and safety similar to data found in the literature.

## RESUMO

**Introdução:**

com o aperfeiçoamento e a ampla aceitação da laparoscopia nas operações colorretais, houve necessidade de treinamento específico dos cirurgiões em formação. Existem poucos estudos avaliando os resultados pós operatórios das colectomias videolaparoscópicas realizadas por médicos residentes e seu impacto na segurança do paciente.

**Objetivo:**

analisar os resultados cirúrgicos e oncológicos das colectomias videolaparoscópicas realizadas por residentes de coloproctologia e comparar com dados da literatura.

**Métodos:**

trata-se de uma análise retrospectiva de pacientes submetidos a cirurgias colorretais laparoscópicas, realizadas por médicos residentes do Hospital das Clínicas de Ribeirão Preto, entre 2014 e 2018. Foram estudadas as características clínicas dos pacientes bem como os principais aspectos cirúrgicos e oncológicos em um período de um ano.

**Resultados:**

analisou-se 191 operações, cuja principal indicação cirúrgica foi adenocarcinoma, a maioria estadio III. A duração média das cirurgias foi 210±58 minutos. Houve necessidade de estoma em 21,5% dos pacientes, principalmente colostomia em alça. A taxa de conversão foi 23%, sendo 79,5% por dificuldades técnicas, e os principais fatores preditores de conversão foram obesidade e acidentes intra-operatórios. A mediana do tempo de internação foi 6 dias. Anemia pré operatória associou-se a uma maior taxa de complicações (11,5%) e reoperações (12%). Houve comprometimento das margens de ressecção cirúrgica em 8,6% dos casos. A taxa de recidiva em um ano foi de 3,2%, e a taxa de mortalidade, 6,3%.

**Conclusões:**

a cirurgia colorretal videolaparoscópica realizada por residentes apresentou eficácia e segurança semelhante aos dados encontrados na literatura.

## INTRODUCTION

Laparoscopic surgery for the treatment of colon and rectum diseases was introduced in the mid-1990s, driven by technological advances and the success of this approach in other gastrointestinal tract procedures^1^ ^,^ ^2^.

Among the advantages of laparoscopy, we highlight the lower endocrine-metabolic response to surgical trauma and, consequently, earlier recovery of digestive tract functions, with the possibility of rapid introduction and evolution of oral diet, thus reducing the length of hospital stay and allowing the patient to return to daily activities in a shorter time when compared with open surgery^3^ ^-^ ^6^.

Currently, laparoscopic surgery has good applicability in the elective surgical treatment of the main colorectal pathologies^7^. Among these, colorectal cancer is the most frequent, as it is the third most prevalent neoplasm in the world^8^ ^,^ ^9^.

With the advancement of minimally invasive colorectal surgery, adequate oncologic resection has become feasible also laparoscopically. When compared with the conventional technique, the safety and oncological results of this access route are equivalent^7^ ^-^ ^10^. In addition to the numerous mentioned benefits, laparoscopy has become increasingly popular among colorectal surgeons and has gradually evolved to become the gold standard in the elective surgical treatment of colorectal pathologies^11^. In large centers, it is estimated that around 59% of colorectal surgeries are performed laparoscopically^12^.

Laparoscopic colorectal surgery, however, is technically complex and requires the acquisition of specific skills to be performed safely^13^, requiring an adequate learning curve from the surgeon. With the development and wide acceptance of laparoscopy for colon and rectal surgeries, the need for appropriate training arose for both staff surgeons and resident physicians^11^.

Some authors tried to demonstrate the minimum number of surgeries for the colorectal surgeon to reach the apex of this learning curve, and it is possible to find in the literature the description of 10 to 200 necessary procedures^14^ ^-^ ^16^, but even today there is no consensus on this cutoff point. Nevertheless, more than just a number, the great discussion within the scope of medical education revolves around the best way to assess proficiency and define objective measures to calculate the real impact of the involvement of resident physicians in the procedures, mainly regarding the results in the short and long term^14^ ^,^ ^17^, since some studies have shown that the involvement of the training resident may be associated with a longer surgical time and a higher morbidity and mortality rate^7^ ^,^ ^18^.

We therefore propose a study with the aim of analyzing laparoscopic colorectal surgeries performed exclusively by resident physicians in a university hospital, evaluating the predictors of unsatisfactory results and comparing the surgical and oncological results with data from the literature.

## METHODS

This is a retrospective study that analyzed the medical records of patients who underwent laparoscopic colectomies between January 2014 and December 2018, carried out after approval by the Ethics in Research Committee of the Hospital das Clínicas, Faculdade de Medicina de Ribeirão Preto, Universidade de São Paulo (HC-FMRP-USP). We assessed operations in which resident physicians of the last years, R4 and R3, acted as first surgeon and assistant, respectively, under the supervision of two assistant physicians of the Coloproctology Division of HC-FMRP-USP. All residents underwent basic laparoscopic training in their first years of residency in general surgery.

We included all patients submitted to laparoscopic surgery for colon and rectum diseases during the period. The oncological principles of resection, such as ligation of the mesenteric vessels at the root and corresponding lymphadenectomies, were applied in all procedures, including the cases of benign disease. We excluded patients who underwent laparoscopy in which the surgery was converted before the main surgical time due to technical difficulties, or in which the attending physician needed to take control of the surgery during the main surgical time.

Patients diagnosed with colorectal neoplasia were staged according to the service’s standard protocol, with colonoscopy with biopsy and CT scans of the chest, abdomen, and pelvis.

From the medical records we collected data on the independent variables identification, registration, race, sex, age, BMI, habits, ASA anesthetic risk classification, preoperative hemoglobin, albumin and total protein levels, tumor marker level (CEA), primary site neoplasm, and disease clinical staging.

We also gathered data on the outcomes duration of surgery, intraoperative accidents, need for stoma construction, postoperative surgical complications, length of stay, early reoperation rate (up to 30 days after the procedure), rate of complete resection of the lesion, late surgical complications, and mortality.

We entered and organized the collected data in a database on the Microsoft Access software and analyzed them with the SPSS (Statistical Package for the Social Sciences) software version 21.0.

We evaluated the variables’ distribution with the Kruskal-Wallis test. For sample characterization and descriptive analysis, we used frequency measures (absolute count and percentage), measures of central tendency (mean or median), and measures of dispersion (standard deviation or interquartile range).

## RESULTS

In the analyzed period, 191 laparoscopic intestinal surgeries were performed. The main clinical characteristics of the patients are detailed in Table 1.

**Table 1:** Clinical characteristics of operated patients.

Feature	Observed value
Sex - n (%)	
Male	90 (47.1)
Female	101 (52.9)
Age (years)	
Mean ± SD^1^	58.9 ± 13.5
Range	18-86
Body mass index (Kg/m^2^)	
Mean ± SD	25.6 ± 4.5
Range	16-39
ASA Rating^2^ - n (%)	
I	32 (16.8)
II	140 (73.3)
III	19 (9.9)
Anemia - n (%)	74 (38.7)
Hypoalbuminemia - n (%)	11 (5.8)
Smoking - n (%)	78 (40.8)
Alcoholism - n (%)	57 (29.8)
Prior radiotherapy - n (%)	13 (6.8)

^*1*^ *SD: standard deviation;* ^*2*^ *Surgical risk classification by the American Society of Anesthesiologists.*

The treatment of colorectal cancer (CRC) was the main surgical indication (n=151/79.1%). Table 2 summarizes the main oncological characteristics of the sample.

**Table 2:** Oncological characteristics of patients diagnosed with CRC undergoing laparoscopic surgery.

Feature	Value
Primary site - n (%)	
Colon	111 (73.5)
Rectum	40 (26.5)
Histological subtype - n (%)	
Adenocarcinoma	149 (98.8)
Leiomyosarcoma	1 (0.6)
Neuroendocrine	1 (0.6)
Tumor size in cm (Mean ± SD^1^)	4.3±1.9
Recovered lymph nodes (Mean ± SD)	14.3±7.3
Undifferentiated tumor - n (%)	6 (4.0)
Presence of desmoplasia - n (%)	15 (9.9)
Mucinous differentiation - n (%)	18 (11.9)
ALI+PNI^2^ - n (%)	71 (47.0)
Free margins - n (%)	138 (91.4)
Tumor staging^3^ - n (%)	
0	5 (3.3)
I	26 (17.2)
II	42 (27.8)
III	67 (44.4)
IV	11 (7.3)
IV	11 (7,3)

^*1*^ *SD: standard deviation;* ^*2*^ *ALI+PNI: angiolymphatic and perineural invasion;* ^*3*^ *Recommended by the Union for International Cancer Control (UICC).*

Rectosigmoidectomy was the main operation performed (n=119/62.3%). Table 3 summarizes the main surgical characteristics. 

**Table 3:** Main surgical results of patients submitted to laparoscopy.

Feature	Value
Duration (Average ± SD1)	210.7 ± 58.2
Conversion rate^2^ - n (%)	44 (23.0)
Need for intestinal stoma - n (%)	41 (21.5)
Intraoperative accident - n (%)	12 (6.3)
Early complication^3^ - n (%)	21 (11.5)
Length of stay in days (median, IQR 4)	6 (5-8)
Early reoperation^3^ - n (%)	23 (12)
Early death^3^ - n (%)	4 (2.1)
Late death^5^ - n (%)	8 (4.2)

^*1*^ *SD: standard deviation;* ^*2*^ *Conversion rate to laparotomy;* ^*3*^ *Occurring within 30 days of the surgical procedure;* ^*4*^ *IQR: Interquartile range;* ^*5*^ *Occurring later than 30 days after the surgical procedure.*

Conversion to open surgery occurred in 44 (23.0%) operations and technical difficulties were the main cause (n=35/79.5%). Other causes for conversion were unspecified vascular injury (n=7/15.9%), ureter injury (n=1/0.5%), and iliac vessel injury (n=1/0.5%). In univariate and multivariate analyses, we observed that obesity, diagnosis of malignant neoplasm with invasion of adjacent organs, and intraoperative accidents were predictive factors for conversion to laparotomy (Table 4).

**Table 4:** Multivariate analysis of risk factors for conversion from laparoscopic to open access route.

Factor	Conversion [n (%)]	p^1^	OR (95% CI)
BMI			
Obese vs non-obese	15 (37.5) x 29 (19.2)	0.016	2.7 (1.2-6.4)
Tumor invasion grade^2^			
T4 x T1-3 tumors	7 (53.8) x 37 (20.9)	0.003	6.3 (1.8-21.6)
Intraoperative accident			
With accident x without accident	9 (75.0) x 35 (19.6)	0.001	11.5 (2.8-46.8)

^*1*^ *Fisher’s exact test; OR: odds ratio; 95% CI: 95% confidence interval; BMI: body mass index;* ^*2*^ *Recommended by the Union for International Cancer Control (UICC).*

Intraoperative accidents occurred in 12 (6.3%) procedures. The main accident was unspecified vascular injury (n=7/58.4%), followed by ureteral injury (n=3/25.0%), iliac vessel injury (n=1/8.3%), and bladder injury (n=1/8.3%). In the univariate analysis, we observed no factors associated with a higher accident rate.

Complications occurred in 21 (11.5%) patients. Intestinal anastomotic dehiscence was the main complication (n=8/38.0%), followed by pelvic abscess (n=2/10.0%), abdominal wall dehiscence (n=2/10.0%), enteric fistula (n=2/10.0%), internal hernia (n=2/10.0%), urinary fistula (n=1/5.0%), early adhesion (n=1/5.0%), ischemia of the small intestine (n=1/5.0%), unspecified vascular lesion (n=1/5.0%), and intestinal stoma necrosis (n=1/5.0%). In the univariate analysis, the presence of anemia was associated with a higher rate of early complications.

Early reoperation occurred in 23 (12%) patients. All patients with early complications underwent surgical intervention. One patient underwent revision of the cavity, with no findings, and another underwent surgical reapproach to correct a ureter injury. Anemia was associated with a higher rate of early reoperations.

The early mortality rate was 2.1% (n=4). The presence of early complications and the need for reoperation were factors associated with early mortality (Table 5).

**Table 5:** Multivariate analysis of factors associated with early mortality.

Factor	Early death n (%)	p^1^	OR (95% CI)
Complications			
Yes x No	4 (19.0) x 0	<0.001	1.2 (1.0-1.5)
Reoperation			
Yes x No	4 (17.4) x 0	<0.001	1.2 (1.0-1.4)

^*1*^ *Fisher’s exact test; OR: odds ratio; 95% CI: 95% confidence interval.*

The mortality rate within one year of surgery was 4.2% (n=8). In univariate and multivariate analyses, we observed that early complications and diagnosis of malignant neoplasm with invasion of adjacent organs were predictors of late mortality (Table 6).

**Table 6:** Multivariate analysis of factors associated with late mortality.

Factor	Late death	p^1^	OR (95% CI)
Complications			
Yes x No	3 (14.3%) x 5 (2.9%)	0.036	6.3 (1.1-36.0)
Tumor invasion grade^2^			
T4 x T1-3 tumors	4 (30.9%) x 4 (2.3%)	<0.001	21.2 (4.0-110.0)

^*1*^ *Fisher’s exact test; OR: odds ratio; 95% CI: 95% confidence interval;* ^*2*^ *Recommended by the Union for International Cancer Control (UICC).*

## DISCUSSION

Currently, laparoscopy is the preferred access route to the abdominal cavity in elective operations for the treatment of benign or malignant colorectal diseases, due to its numerous benefits. Despite the advantages and dissemination of the technique in clinical practice, until the present study, little was known about the performance of training physicians, particularly in our country.

In our service, until 2018, resident physicians participated, on average, in 30 laparoscopic colorectal surgeries per year, totaling 60 procedures in the training period. This number can be considered adequate for the formation of the learning curve of colorectal surgeons when compared with series of published cases, which suggest that performing 40 or more surgeries of the colon and rectum by laparoscopy guarantees skill and comfort for training physicians^15^. Some studies, however, have shown that operative experience and the learning curve can be influenced not only by the number of operations, but also by factors such as patient selection and the procedures’ complexity^7^ ^,^ ^10^ ^,^ ^18^ ^,^ ^19^.

Regarding the duration of the procedures, the average intraoperative time was longer than that described by most published studies, especially those that compared the performance of resident physicians with that of assistants in colorectal laparoscopic surgeries. Mehall et al. compared the surgical results of patients operated on by both groups, showing a longer surgical time in the group operated by residents; however, the degree of intraoperative bleeding, as well as conversion rates and major complications between the two groups, were similar. They therefore concluded that the increment in intraoperative time would not increase the risk of complications^20^. The high surgical times observed in the present study were longer than those found in other studies, and can be explained by the random selection of patients, including longer procedures such as laparoscopic total colectomy (in the case of synchronous colorectal neoplasms, for example) and because it is the Service’s initial experience with training residents in colorectal laparoscopic surgery.

Champagne et al. discussed the need to evaluate the best method of training resident physicians to build a good learning curve in laparoscopic colorectal surgery, and concluded that, regardless of the technique used for this purpose, what determines training success is the ability of the resident physician to complete the surgery successfully without causing harm to the patient, independent of intraoperative time^11^.

Conversion from laparoscopic surgery to the conventional open technique is indicated whenever the surgeon determines that patient safety or surgical dissection may be compromised. In this analysis, the conversion rate from laparoscopic surgery to the open approach was in line with the literature, in which most studies mention a rate between 20 and 30%. More than 2/3 of these conversions were due to technical difficulties. Other indications for conversion were iatrogenic injuries identified intraoperatively.

Obesity, locally advanced tumors, and intraoperative accidents were predictive factors for conversion to laparotomy. Despite being cited as predictive factors in the literature, in our study some factors that could increase the difficulty of dissection were not statistically significant, such as tumor size in extension, previous radiotherapy, and initial diagnosis.

In more than half of obese patients, laparoscopic colorectal surgery was feasible, without conversion or complications. However, a conversion rate above 35% in the sample of obese patients is high and needs to be taken into account, since 55.1% of patients undergoing colorectal laparoscopic surgery were classified as overweight or obese. Homma et al. suggested that high BMI would be an independent factor for conversion and intraoperative complications. In the present study, laparoscopic colorectal surgery was performed in more than 60% of the patients, without complications or need for conversion to the open approach. Even so, the conversion rate in obese individuals was higher, in agreement with the literature^21^. To reduce this percentage, Parker, Homma, and Miskovic suggested selecting the cases that should be operated on during the learning process^21^ ^-^ ^23^.

The immediate identification of an accident still during the performance of a surgical procedure reduces the chances of postoperative complications, which could become more serious if identified late. Intraoperative accidents end up indicating the conversion of surgery to the open approach, especially during the learning curve, when the resident physician still does not have sufficient skills to solve complications by laparoscopic approach. In this study, there was conversion in 75% of the cases in which accidents were identified during the intraoperative period, with immediate resolution during the same surgical time. We analyzed several factors to assess whether there was an association with an increase in the incidence of accidents, but none was statistically significant. The main accidents described in this study were vascular, bladder, and ureteral injuries. Most published papers mention similar accidents^24^.

Kirchhoff et al. found a rate of intraoperative complications of 7.4%, reporting, in addition to bleeding and urinary tract injuries, intestinal injuries and problems with making the anastomosis, as well as 13% of anesthetic complications. Unlike what we found in the present statistical data (absence of direct association between risk factors and occurrence of accidents), this group described that advanced age, comorbidities, male sex, and diagnosis of neoplasia would be predictive factors for the occurrence of intraoperative accidents^25^.

The success of an anastomosis is related, among other factors, to good vascularization and absence of tension, in addition to the surgeon’s degree of experience. When one of the factors is compromised, the possibility of making a protective stoma is considered, with early closure programmed, necessary in 21.5% of the patients in the sample. Other authors have found similar rates, with protective loop ileostomy generally preferred by several authors^26^. In our institution, however, loop colostomy is the most used option.

We defined early complications as those occurring up to 30 days after surgery. Such complications occurred in 11.5% of the patients, the main one being anastomotic dehiscence. In addition to this complication, other authors mentioned surgical site infection and late bleeding, and reported that preoperative anemia had a significant influence on intraoperative complications, but with little influence on postoperative morbidity^27^.

In the present study, however, preoperative anemia was the only statistically significant factor associated with early postoperative complications, present in 2/3 of patients who evolved with complications within 30 days after the procedure. Other factors evaluated were hypoalbuminemia and previous radiotherapy, but the statistical analysis did not show a direct association between these factors and the increase in postoperative complications.

All patients who had early complications were reapproached, including the eight patients who evolved with anastomotic dehiscence, which is the main indication for surgical revision. In all, 12% of patients underwent a new surgical procedure within one month of the initial surgery. In addition to the patients who evolved with the early complications described, one patient with an intraoperatively unidentified ureteral lesion was reoperated, and evolved with a urinary fistula, and another patient who evolved with clinical worsening requiring a cavity second look. Preoperative anemia was also the main factor associated with early reoperations. The other factors analyzed did not show statistical significance.

Regarding length of stay, we found that, on average, patients needed to remain in the hospital environment for about six days, shorter than the averages described in the literature, and close to the statistics of surgeons with good experience in laparoscopy. Del Rei et al. recorded a longer hospital stay, around nine days^28^. Kirshhoff et al. reported an average time of 10.5 days^25^.

Most studies that compared the results of surgeries performed by the training surgeon and the assistant surgeon reported that the length of stay between the two groups was similar^29^. Gongun et al., in turn, reported that, though the hospitalization time of the patients submitted to laparoscopic colorectal surgery by the group of residents was slightly longer than in the control group, this difference could increase the hospital costs of treating such patients, causing an important financial impact, and a cost-benefit analysis should be made^30^.

The analysis of mortality in this population undergoing laparoscopic colorectal surgery performed by resident physicians is essential to define the safety of this procedures for the patient. The main factors associated with early mortality (within 30 days after surgery) were the presence of postoperative complications and the need for early reoperation, present in the clinical evolution of all patients who died, and the rate found was 2.1%, similar to that reported by the national multicenter study published by Campos et al., up to 3.2%, and to the studies cited in a Brazilian study (0.7 to 2.1%)^31^.

We carried out a retrospective follow-up of the patients in this study for a period of one year, showing a late mortality rate of 4.2%. Statistically, the factors that were associated with this poor prognosis were the presence of postoperative complications and advanced staging of colorectal cancer at diagnosis.

We recorded no deaths between 30 days and one year after surgery among patients with stages I and II, only in patients with stages III and IV. The late mortality rate in this group was much higher than the reported in the literature, almost 31%, compared with 2% in the group of patients with earlier disease. The degree of tumor invasion (“T” staging) was an important predictor of late mortality, showing an increased risk in the group of patients with locally advanced disease, with invasion of adjacent organs, in agreement with the article by Shootman^32^.

## CONCLUSION

Performing laparoscopy in a university environment was technically safe, with acceptable complications rates and without a significant increase in patient morbidity and mortality, similar to data in the literature.
